# Co-Administration of Injected and Oral Vaccine Candidates Elicits Improved Immune Responses over Either Route Alone

**DOI:** 10.3390/vaccines8010037

**Published:** 2020-01-21

**Authors:** Celine A. Hayden, Danilo Landrock, Chiung Yu Hung, Gary Ostroff, Gina M. Fake, John H. Walker, Ann Kier, John A. Howard

**Affiliations:** 1 Applied Biotechnology Institute, Cal Poly Tech Park, San Luis Obispo, CA 93407, USA; chayden@appliedbiotech.org (C.A.H.); gfake@appliedbiotech.org (G.M.F.); 2 Department of Veterinary Pathobiology, College of Veterinary Medicine, Texas A & M University, College Station, TX 77843, USA; dlandrock@tamu.edu (D.L.); akier@cvm.tamu.edu (A.K.); 3 Department of Biology, University of Texas San Antonio, One UTSA Circle, San Antonio, TX 78249, USA; chiungyu.hung@utsa.edu; 4 Program in Molecular Medicine, University of Massachusetts Medical School, 373 Plantation St. Biotech 2, Suite 113, Worcester, MA 01605, USA; gary.ostroff@umassmed.edu; 5 Department of Statistics, California Polytechnic State University, San Luis Obispo, CA 93407, USA; jwalker@calpoly.edu

**Keywords:** subunit vaccine, mucosal, maize oral vaccine, plant vaccine, bioencapsulation, immunogenicity, supercritical fluid extraction

## Abstract

Infectious diseases continue to be a significant cause of morbidity and mortality, and although efficacious vaccines are available for many diseases, some parenteral vaccines elicit little or no mucosal antibodies which can be a significant problem since mucosal tissue is the point of entry for 90% of pathogens. In order to provide protection for both serum and mucosal areas, we have tested a combinatorial approach of both parenteral and oral administration of antigens for diseases caused by a viral pathogen, Hepatitis B, and a fungal pathogen, *Coccidioides*. We demonstrate that co-administration by the parenteral and oral routes is a useful tool to increase the overall immune response. This can include achieving an immune response in tissues that are not elicited when using only one route of administration, providing a higher level of response that can lead to fewer required doses or possibly providing a better response for individuals that are considered poor or non-responders.

## 1. Introduction

Infectious diseases continue to be a significant cause of death despite the identification of highly efficacious vaccines. Several factors play a role in the ability of a vaccine to provide immunity. For example, while the antigen may elicit an immune response in a certain tissue, it may not be at the site required for protection. To illustrate this point, many injected vaccines elicit little or no mucosal antibodies which can be a significant problem since mucosal tissue is the point of entry for 90% of pathogens. Even in cases where the antigen can induce the required response, factors such as cost, cold chain, aversion to needles, and inaccessibility of doctors lead to non-compliance.

A case in point is hepatitis B. Despite the availability of an effective parenteral vaccine for over 20 years, hepatitis B virus (HBV) remains an important problem, with 240 million chronically infected patients worldwide [1]. The present recommendation for the vaccine consists of injecting a primary dose of the HBV surface antigen, HBsAg, followed by two boosting doses. Although seroconversion occurs in greater than 90% of the general population using commercialized vaccines when the three-dose regimen is followed [2], many individuals, including first responders who are most likely to be exposed do not complete the three-dose regimen [3,4]. In addition, there are specific segments of the population that are poor or non-responders. Among them are the elderly, obese individuals, HIV-positive patients, and individuals with celiac disease, irritable bowel disease, Down syndrome, or chronic kidney disease [5,6,7,8,9,10,11,12,13,14,15]. Therefore, a more effective vaccine may alleviate some of these problems.

In an attempt to resolve these deficiencies with the commercial vaccine we developed an oral vaccine candidate that greatly improves the immune response at mucosal sites [16], which are the tissues that are traditionally primary sites of infection. It is thought that the low moisture, high carbohydrate concentration, and high levels of protease inhibitors in the vaccine favor survival of the vaccine through the upper gastro-intestinal tract [17,18,19]. For hepatitis B, this has the potential to be particularly beneficial for adults where the major route of infection is through sexual transmission. In addition, an oral vaccine would be easier to administer and could increase compliance in populations that historically show non-compliance with the HBsAg three dose regimen, such as hemophiliacs [20], at-risk youth [21], transient populations [22], persons engaging in high-risk sexual activity [23,24], and healthcare workers [4]. On a global scale, an oral alternative could provide a low-cost, heat-stable alternative to parenteral vaccines [2,25] and therefore improve coverage in remote or resource-poor areas that cannot afford the infrastructure for reliable cold storage, needle administration, and waste disposal.

While these arguments present a salient case for using the oral vaccine to elicit a mucosal response, a protective response is determined using the WHO International Standard which relies on serum antibody titers. Therefore, we asked the question whether there was a way to combine the oral and injected routes of administration to achieve an improved overall immune response, including serum antibodies. Testing multiple routes of administration has been shown to improve the response for other vaccine candidates [26,27] when given at separate times. However, we are unaware of any study that administered both injected and oral candidates at the same time. Our objective, therefore, was to evaluate the response of co-administration of the vaccine candidate by multiple routes to assess whether we could induce an additive or synergistic response. Our results presented below suggest that this is the case for HBsAg.

Another example where an improved immune response may be helpful involves the *Coccidioides* species responsible for Valley Fever. This pathogen is endemic to the soils of the arid and semi-arid regions of the southwestern United States as well as other semi-desert areas of the Americas. Sixty percent of infections are asymptomatic and the remaining 40% result in pulmonary disease that mimics flu-like symptoms [28]. Encouragingly, individuals who have recovered from symptomatic Valley Fever achieve life-long immunity to *Coccidioides* infections [29,30]. Unfortunately, symptomatic infections lead to chronic disease in 5% of cases and extrapulmonary dissemination of the fungi in 1% of cases [31]. An estimated 150,000 new infections occur each year in the United States [31] and the incidence of reported cases has increased 8-fold since 1998 [32]. For the significant population base that lives in, trains in, or travels to these desirable warm weather areas, a vaccine would be highly beneficial. In addition, long-term protection via vaccination is likely to be achieved since natural infections with *Coccidioides* provide life-long immunity [29,30].

Providing adequate protection for fungal pathogens is problematic as evidenced by the fact that there are no fungal vaccines on the market today. Several approaches in the literature have been used to potentiate the immune response for subunit vaccines. One approach that has shown promise is the use of glucan particles as an antigen presenting cell (APC) receptor-targeted adjuvant delivery system to enhance an immune response [33,34]. There is strong evidence that a cell-mediated response is required for protection against *Coccidioides*, and glucan-chitin particles (GCPs), produced from a non-pathogenic yeast *Rhodotorula mucilaginosa*, have been shown to be an effective adjuvant-delivery system to stimulate a protective Th1 and Th17 mixed response against *Coccidioides* [35].

We recently tested the potential for oral delivery of the antigen to improve the cell-mediated response. Orally delivered antigens in combination with GCPs showed a slight, but not statistically significant, improvement of the cell mediated immune response [36]. The results were inconclusive due to saturation of the assay. We wanted to follow up on this by co-administering the antigen with injected GCPs and orally delivered antigen. Co-administration using an oral subunit has not previously been shown. However, there are reports of co-administration with oral- or nasal delivered nucleic acid vaccine candidates coupled with injections [37,38,39,40]. Preliminary data here suggest our oral-parenteral coadministration may be a more effective route for providing protection.

To our knowledge, this is the first report of using co-administration with an oral subunit vaccine to enhance an immune response. This procedure can provide a new tool to improve immunization for non-responders, reduce the number of doses required for immunization, or provide a more effective immune response across multiple tissues thereby providing greater protection.

## 2. Materials and Methods

### 2.1. Maize Material

Maize plants containing the HBG DNA construct expressing hepatitis B surface antigen (HBsAg) in tandem duplicate plant transcription units were grown and selected for highest expressing lines over seven backcrosses to elite parental Stine inbreds 16038 and MBS5411 [26]. The HBG 16038-introgressed line was selfed to create a homozygous line and crossed to a heterozygous MBS5411 line to create hybrid seed. Hybrid seed was planted and HBsAg grain was harvested.

Maize plants containing the VFG DNA construct [25] expressing a recombinant *Coccidioides* Ag2 protein fused to a dendritic cell-targeting peptide (DCpep), were backcrossed to maize elite parental inbred line 16038. Control germ (G909) was obtained from the Grain Processing Corporation (Muscatine, IA, USA).

### 2.2. Seed Processing

HBsAg grain was fractionated using a dry degerming method with a pilot-scale custom degermer. The germ fraction was ground using a GlenMills grinder, passed through a 20-mesh sieve, and lipids removed as previously described using CO_2_ supercritical fluid extraction (SFE) [16]. In brief, a 5L SFT-250 (Supercritical Fluid Technologies, Newark, DE, USA) was maintained at 350 bar, with a target vessel temperature of 35–40 °C (maximum of 45 °C), and a flow rate between 10 and 40 SCFH until 80%–86% of the oil was removed in the HBsAg germ and until 70% was removed in the control germ.

Maize seed material from the VFG backcross was ground and passed through a 20-mesh sieve before being incorporated into wafers.

### 2.3. Vaccine Preparation

Wafers containing the antigens were made as described previously [16]. In brief, each wafer consisted of 2.5 g ground maize material (delipidated HBsAg germ, Ag2 material or control material), 1.25 g baker’s sugar (C & H), and either 0.4 g of water (HBG and Ag2 wafers) or 0.8 g of water (control wafers). Ingredients were mixed manually, wafers formed using a manual press, and molded wafers dried in a vacuum oven (VWR 1430, VWR, Radnor, WA, USA) at 50 °C, and 23.5–24.5″ Hg of vacuum pressure applied until >90% of the added water was removed.

Injected doses of maize-purified Ag2 were prepared in GCPs (Ag2), as previously described [36]. Briefly, Ag2m was purified on an immunoaffinity column and 0.1 μg of Ag2 was loaded into GCPs along with mouse serum albumin. Injected doses of bacterium-purified Ag2 were prepared in GCPs (Ag2b), as previously described [41], with an Ag2 dose of 1.0 μg. The discrepancy in bacterial and maize Ag2 injected dose was consciously adopted in order to remain within timelines.

### 2.4. HBsAg Mouse Vaccine Trial

Pathogen-free BALB/c female mice were randomly assigned to treatments groups, with each group consisting of eight mice. All groups were injected with an intra-muscular dose of HBsAg on day 0 and group 3 mice were also fed an oral dose of HBsAg wafers on day 0. Group 1 mice were injected on days 0, 98, and 112 (I/I/I), group 2 mice were injected on day 0 and orally fed wafers on day 98 and 112 (I/O/O), group 3 mice were injected and orally fed HBsAg material on days 0 and 98, but no third dose was administered (IO/IO), while group 4 mice were injected on day 0 and fed two subsequent control wafer doses on days 98 and 112 that contained no HBsAg. Each mouse consumed a mean estimated 2.0 mg HBsAg per oral dose.

### 2.5. HBsAg and Ag2 Quantification

Extraction and detection of the HBsAg subunit or the Ag2 protein was carried out using a custom ELISA, as previously described [16,36,42]. Briefly, ground maize material was extracted in PBS with 1% TritonX-100 and applied to a custom sandwich ELISA for detection of antigen concentration.

### 2.6. Hepatitis B Vaccine Mouse Study

Individually-housed pathogen-free female BALB/c adult mice were randomly assigned to one of four treatments, with eight mice per treatment. Injected doses consisted of intra-muscular administration of 0.25 µg of Recombivax (lot#MO13639, Merck, Whitehouse Station, NJ, USA) and oral doses consisted of feeding, *ad libitum*, two 3.75 g wafers over three days. Uneaten wafer was removed from the cages 24 h after placement of the two wafers in the cage. An estimated 2.0 mg HBsAg was delivered per oral dose, as calculated using the mean HBsAg concentration in wafers and the mean consumption of mice fed the oral HBsAg dose. The first treatment group received injected doses at day 0, 98, and 112 (I/I/I). The second group received a primary injected dose and two oral boosting doses on the same schedule (I/O/O). The third group received a co-administered injected/oral primary dose and a single co-administered boosting dose, but no third dose (IO/IO). The final group received a single injected primary dose and two control oral boosting doses (I/C/C). Immunization schedule was selected based on previous optimization studies [16,36,43].

Serum was collected (i) preceding the primary dose (pre-immune), (ii) once a month until the initial immune response showed evidence of decreasing, (iii) directly preceding the first boosting dose (week 15), and (iv) every two weeks until the end of the study. Fecal material was collected (i) preceding the primary dose (pre-immune), (ii) directly preceding the first boost, and (iii) twice per week until the end of the study. Vaginal washes were collected (i) preceding the primary dose (pre-immune), (ii) at week 15, preceding the first boost, (iii) at week 19, 2 weeks after the timing of the second boost, and (iv) at week 20, preceding the terminal bleed. Collection and storage of serum and fecal pellets were as described previously [43]. Collection of the vaginal washes was done with 0.05 mL of sterile PBS flushed 4 to 5 times into the vaginal canal using a plastic pipet tip, collected, centrifuged, and the supernatant stored at −20 °C until used on ELISA assays after a single thaw cycle.

**Figure 1:**
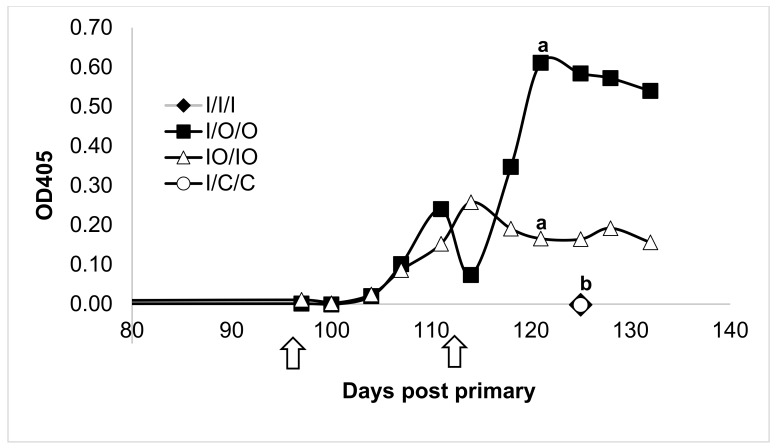
Fecal IgA response to injected, oral, or co-administered injected/oral HBsAg treatments. Mice were administered (i) three injected doses (I/I/I), (ii) injected primary followed by two oral boosts (I/O/O), (iii) co-administered injected and oral dose for the primary and first boost (IO/IO), or (iv) an injected primary followed by two control oral boosting doses (I/C/C). Black arrows represent times of boosting. Only oral treatments elicited a detectable response. Letters above the trendlines represent statistical analysis of the responses. Treatments with different letter designations are significantly different while treatments with the same letter designation fail to show a statistically significant difference.

**Figure 2:**
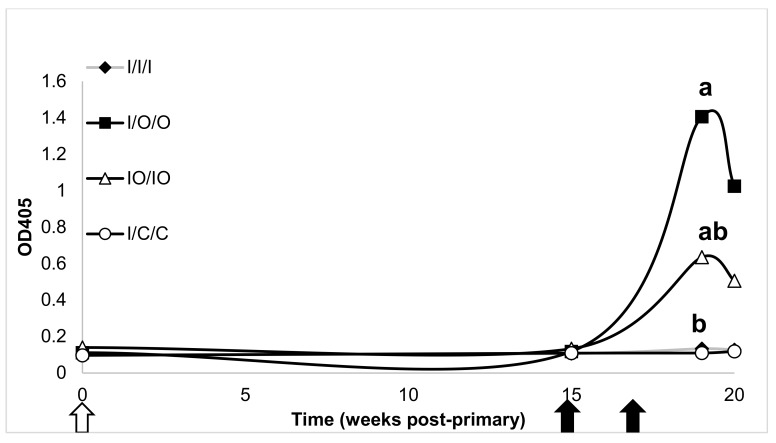
Vaginal IgA response to injected, oral, co-administered injected/oral or control treatments of HBsAg. Treatment groups as in Figure 1. The white arrow represents timing of the primary dose and the black arrows timing of boosting doses. Antibody levels were detected using an ELISA and statistical significance was assessed using ANOVA. Statistical analysis as in Figure 1.

**Figure 3:**
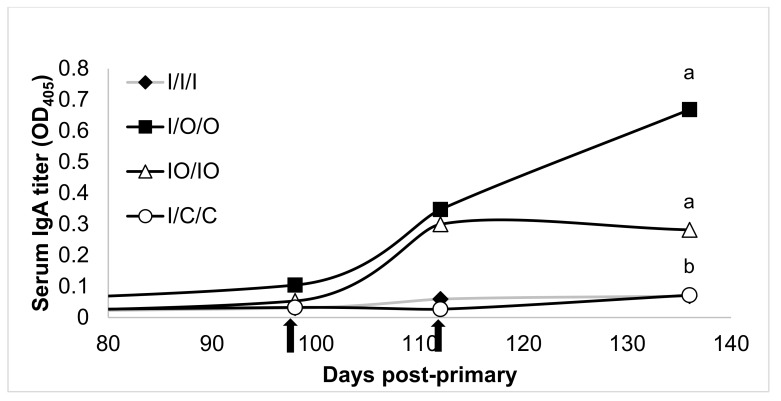
Serum IgA response to injected, oral, co-administered injected/oral or control treatments of HBsAg. Treatment groups, arrows, and statistical analysis as in Figure 1.

**Figure 4:**
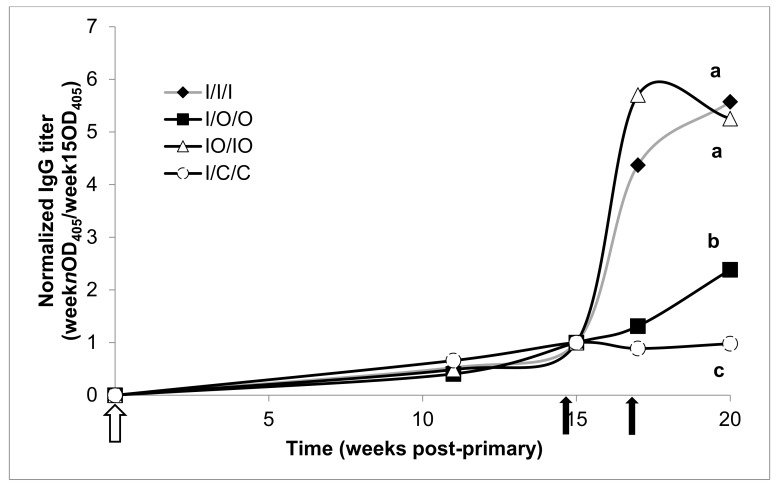
Serum anti-HBsAg IgG geometric means normalized to pre-boost titers. Treatment groups, arrows, and statistical analysis as in Figure 2.

Mouse treatments were compared on each response variable using one-way analysis of variance (ANOVA). Normalized mean titers were assessed at the terminal bleed (O.D. at week 20/O.D. at week 15). Because ANOVA assumes that each treatment has equal variability with respect to the response variable. All of the response variables in the study showed some signs of unequal variation. To correct for unequal variation among response variables, each response variable was transformed using the base 10 logarithm. A 5% individual significance level was used for the ANOVA, and means for each treatment were separated using Tukey’s HSD procedure with a 5% significance level. Treatments with different letter designations (e.g., ‘a’, ‘b’, ‘c’) in Figure 1, Figure 2, Figure 3 and Figure 4 are significantly different while treatments with the same letter designation fail to show a statistically significant difference.

### 2.7. Anti-HBsAg Antibody Detection in Mice

Fecal IgA, Serum IgA, Serum IgG, and total Ig (mIU/mL) were determined as previously described [16] with the exception that fecal pellets were not treated with protease inhibitor. Vaginal IgA and IgG were detected by the same assay used for serum samples, with the exception that vaginal washes were diluted 1/50 and 1/30, respectively.

### 2.8. Coccidioides Vaccine Mouse Study

Each treatment group consisted of 10 BALB/c mice, each containing 5 males and 5 females. Mice were fasted overnight before each dose in order to ensure maximal uptake of oral vaccine, administered on days 0 and 14. The oral wafer dosing was conducted as for the HBsAg mouse study [16]. On each dose day, group 1 mice were treated with a subcutaneous injected dose of Ag2b, group 2 mice received an injected dose of Ag2m, group 3 mice were injected and fed wafers of Ag2m, and group 4 mice were injected intramuscularly with control GCPs and fed control wafers containing no Ag2. On day 42, mice were challenged with ~100 live *Coccidioides* arthroconidia. This immunization schedule was selected based on previous optimization studies that indicated this regimen was in the range to show if there was a difference in the health and immune response of the mice upon treatment with the vaccine candidates [1,2,3].

### 2.9. Analysis of CFUs and Weight Change

The lung fungal burden was assessed at day 56, 14 days after challenge. The number of colony forming units (CFUs)/lung was evaluated and statistically significant differences assessed using the Mann-Whitney *U* test, as previously described [44]. Mice were weighed directly preceding the live challenge (0 dpc), and then at 7 dpc, and 10 dpc. The change in weight compared to 0 dpc was calculated and the Student *t*-test was used to determine whether statistically significant differences were apparent compared to the control group.

## 3. Results

### 3.1. HBsAg Vaccine Production

Hybrid seed expressing HBsAg at a level of 150 mg/kg (SD +/− 4 mg/kg) was harvested from the field and 95 kg was fractionated into germ and endosperm using a customized dry degerming process for small volumes. Approximately 18 lbs of germ were produced with an expression level of 404 mg/kg (SD +/− 42 mg/kg), realizing a 2.7-fold increase in HBsAg concentration. Removal of the lipids using supercritical CO_2_ extraction brought the concentration to 481 mg/kg (SD +/− 70 mg/kg).

This delipidated, ground maize material was then formulated with sugar and water, formed into wafers, and dried in a vacuum oven to produce wafers for the mouse trial. The final concentration of HBsAg in the sugar-containing wafers was measured to be 382 mg/kg (SD +/− 29 mg/kg), consistent with sugar comprising 33% of the wafer and HBsAg flour comprising 67%.

### 3.2. Mucosal Response

Consistent with previous results, when the sole route of administration of HBsAg was by injection (Figure 1, I/I/I) there was no detectable mucosal response and it was not statistically different than the control treatment (I/C/C). In contrast, both treatment groups that were orally administered wafers produced statistically significantly increased fecal IgA responses. While both oral treatments received two oral doses, the treatment receiving the primary injection and two oral boosts (I/O/O) appeared stronger than the treatment receiving the co-administration of injections with two oral doses (IO/IO).

Vaginal IgA to HBsAg was also assessed in treated mice (Figure 2). The strongest response was elicited when an injected dose was followed by two oral boosting doses (I/O/O). As with fecal IgA, the vaginal IgA in the co-administered injected/oral treatment was elevated (IO/IO), but at a lower level than the I/O/O treatment. Also in keeping with the fecal IgA results, the control group (I/C/C) and the all-injection group (I/I/I) did not produce any detectable antibodies at this location.

No statistically significant difference was detected in vaginal IgG for any of the treatments indicating that vaginal IgG is not induced at high levels in either injected or oral administration of HBsAg.

### 3.3. Serum Response

Serum IgA responses to HBsAg administration were similar to the mucosal responses in the oral HBsAg treatment groups, in that only orally administered treatments elicited detectable levels of the antibody. Mice treated with co-administered injected/oral doses (IO/IO) reached a maximal level after the first boost and was relatively well maintained over time in the absence of a second boost, while the response in mice orally boosted twice (I/O/O) continued to increase the serum IgA titer, as expected, after the second boost (Figure 3).

**Figure 5:**
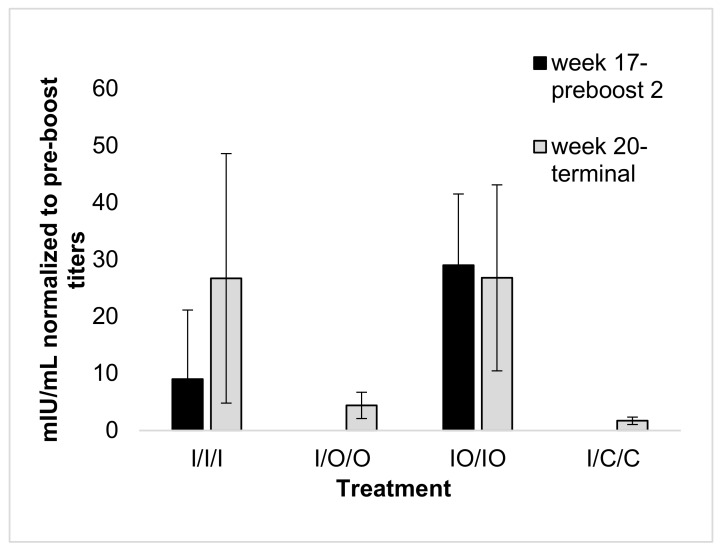
Geometric mean titer of serum anti-HBsAg Ig (mIU/mL) normalized to pre-boost titers with standard error. Mouse treatment groups as in Figure 1. All mice were tested for mIU/mL at the terminal bleed (week 20, day 133), treatments I/I/I and IO/IO were tested at week 17 (day 112), directly preceding the second boost, and all titers were normalized to pre-boost titers at week 15.

In contrast to serum and fecal IgA, injection alone treatments produced strong serum IgG and normalized total Ig (mIU/mL) responses after three doses (Figure 4 and Figure 5), with mIU/mL reaching 51,827 mIU/mL for the IO/IO treatment, and 39,785 mIU/mL for the I/I/I treatment. The two co-administered injected/oral doses gave a statistically equivalent response compared to mice treated with three doses by injection. In addition, the IO/IO week 15 administration was effective in stimulating an IgG response which was sustained through to the week 20 terminal bleed.

### 3.4. Coccidioides Mouse Study

In order to further investigate the benefits of co-administration of oral and injected vaccines, a second study was conducted, consisting of antigens designed to protect against coccidioidomycosis. A *Coccidioides*-derived recombinant antigen, Ag2, was either produced in a bacterial culture (Ag2b) or in a maize seed expression system (Ag2m), and purified forms were administered to mice.

**Figure 6:**
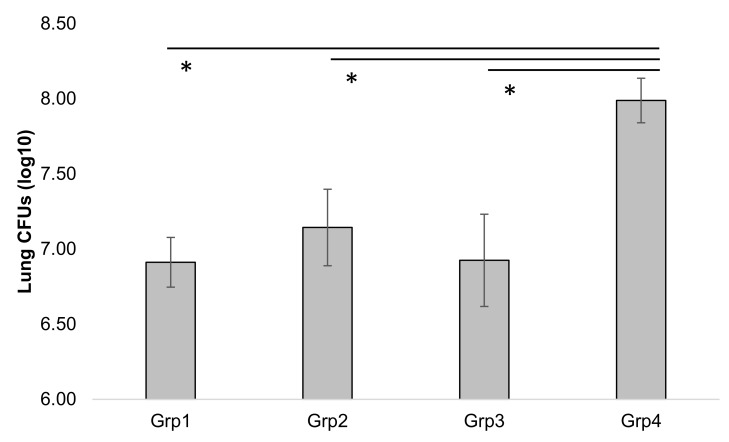
Lung CFUs in mice 14 days post-challenge, with standard error. Statistically significantly lower levels of colonies were observed in all Ag2 treatment groups (Grp 1 to 3) compared to the control group (Grp4; *p* < 0.05), as represented by asterisks (*). Each group received two doses (days 0 and 14) of injected Ag2b (Grp1), injected Ag2m (Grp2), co-administered injection with orally fed Ag2m wafers (Grp3), or control injection with control Ag2m wafers (Grp4).

For injected doses, Ag2b and Ag2m antigens were purified and administered in glucan-chitin particles (GCPs). For oral doses, the bacterial antigen was loaded into GCPs and administered by gavage, while the maize-produced antigen was delivered in wafers made from ground maize material and consumed *ad libitum* by the mice. Treatments consisted of two doses of injected Ag2b (Group1) or Ag2m (Group 2), co-administered injected Ag2m and *ad libitum* orally consumed Ag2m wafers (Group 3), or a control group receiving an injected control GCP particle and control wafer (Group 4). It is estimated that the Ag2m oral dose was approximately 1.5 mg. These mice were then challenged with live *Coccidioides* arthroconidia and assessed for colonization in the lungs 14 dpc (Figure 6).

At 14 dpc, there was a significant decrease in spore lung burden for all treatment groups receiving Ag2, relative to the control treatment group. The immune response did not show any statistical difference in contrast to the previous report when a 4-dose regimen was used.

**Figure 7:**
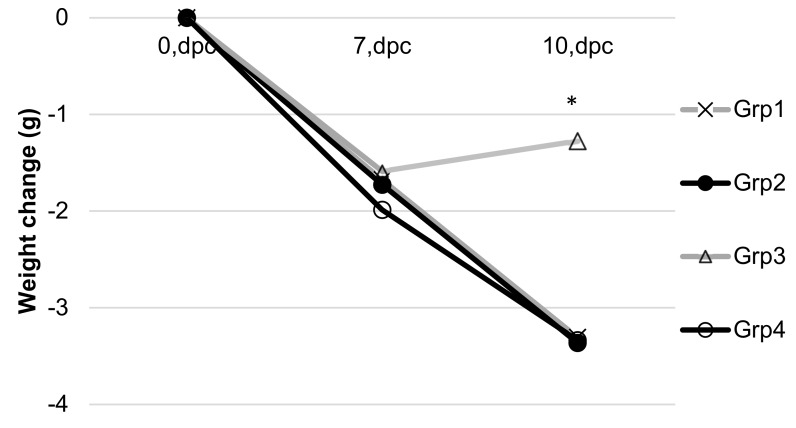
Weight change following challenge with live *Coccidioides* arthroconidia. Asterisk (*) indicates a statistically significant difference between Grp 3 and Grp 4 at 10 dpc (*p* < 0.05). Treatment groups are as described in Figure 6.

A change in weight over time was also evaluated as another metric of mouse health. In this case, only the treatment in which mice were co-administered maize wafers and injected GCPs (Group 3) showed a statistically significantly improved response relative to the control group (Group 4) with Group 3 mice demonstrating an increase in mean weight 10 days post challenge (dpc) (Figure 7). It is also of note that, as of 14 dpc, one mouse died in each of Groups 1, 2, and 4, while no mice died in the oral administration group (Groups 3). While this is not statistically significantly different, it may indicate that oral treatment provides a longer survival profile, but additional studies with a longer post-challenge timeline will be needed to resolve whether these differences can be attributed to oral treatments. No gender differences were observed in the study.

## 4. Discussion

In this study, we compared the immune responses in mice of co-administered oral and injected vaccines for HBsAg and *Coccidioides* Ag2 antigens prepared in maize.

Total serum antibody, when evaluated by the WHO International Standard, is the accepted method to assess whether a protective response has been raised against hepatitis B. When this method was applied to the assessment of the serum immune response following co-administration, the results indicate that two doses of co-administered vaccine were equivalent to three doses of the injected commercial vaccine. Saving one dose can be significant in terms of cost and logistics when immunizing the general public. This may also indicate that a more robust response is possible that can lead to better protection for poor or non-responders to the injected vaccine. In addition to the serum response, the IgA response using the oral route of administration gave a robust IgA response in both serum and mucosal tissues. This may be beneficial for pathogens that enter through the mucosal route, including hepatitis B transmission in adults.

Whereas injected doses did not elicit detectable IgA titers when used as the sole route of administration, when followed by two oral doses, the IgA titers were higher than that obtained with two oral doses co-administered with the injected dose. This may indicate that while the injected dose does not elicit a mucosal response, it may prime the immune system to be more receptive to the oral dosing.

In addition to improving the humoral response, co-administration may also increase the cell-mediated response. Previous results suggested that co-administration may improve the cell-mediated response when a 4-dose regimen was used which correlates with protection of mice after challenge with *Coccidioides* [36]. Here we observed reduced loss of weight for mice when co-administering the vaccine candidate via oral and injected routes with only a 2-dose regimen. Follow-up studies will be done to investigate the optimal number of doses for protection and correlation with the immune response. It would also be beneficial to investigate the effectiveness of various oral-compatible adjuvants, such as the *E. coli* heat-labile toxin (LT) and polysaccharide adjuvants such as Advax^TM^.

Taken as a whole, the data presented here demonstrate that co-administration can provide a very useful tool to increase the overall immune response. This can include, achieving an immune response in tissues that are not elicited when using only one route of administration, providing a higher level of response that can lead to fewer required doses or possibly providing a better response for individuals that are considered poor or non-responders. From a practical perspective, using the maize wafers for oral delivery also provides a low cost and low intrusive method that can easily supplement injected vaccines.
